# Age, creatinine, and ejection fraction score is a risk factor for acute kidney injury after surgical aortic valve replacement

**DOI:** 10.1080/0886022X.2024.2444401

**Published:** 2025-01-13

**Authors:** Tingting Wu, Rui Li, Jing Chen, Xiaqiu Tian, Ran Zhang, Xiaotong Hou

**Affiliations:** a Center for Cardiac Intensive Care, Beijing Anzhen Hospital, Capital Medical University, Beijing Institute of Heart, Lung and Blood Vessel Diseases, Beijing, China; b Department of Health Care, China-Japan Friendship Hospital, Beijing, China

**Keywords:** Acute kidney injury, surgical aortic valve replacement, risk factor, ACEF score

## Abstract

**Background:**

The incidence of acute kidney injury (AKI) increases after surgical aortic valve replacement (SAVR). This study aimed to characterize the risk factors of AKI after SAVR.

**Methods and Results:**

We conducted a retrospective registry study based on data from 299 consecutive patients undergoing SAVR. At 48 h after SAVR, 41 patients developed AKI. There was a significantly higher prevalence of older age, higher body mass index (BMI), and diabetes mellitus in the AKI group. Previous use of angiotensin-converting enzyme inhibitors/angiotensin receptor blocker (ACEI/ARB) and β-blocker, intake and output volumes within 24 h, mechanical ventilation time, length of intensive care unit and hospital stay, baseline creatinine, baseline, 24 h, and 48 h estimated glomerular filtration rate were strongly associated with the incidence of AKI. BMI >24, history of hypertension, use of ACEI/ARB and β-blocker, and mechanical ventilation time were associated with AKI. Univariate logistic regression indicated that overweight, hypertension, use of ACEI/ARB and β-blocker, and mechanical ventilation time were associated with AKI. Notably, the ACEF score was an independent predictor of AKI. The receiver operating characteristic curve was employed to assess the ACEF score for predicting AKI and the best cutoff was 1.1. After dividing ACEF into quartiles, each one-unit increment in ACEF led to a 2.27-fold risk in the incidence of AKI among patients.

**Conclusions:**

AKI is a generalizable phenomenon occurring frequently after SAVR. The ACEF score is an independent predictor of AKI among patients undergoing SAVR.

## Instruction

With a rapid increase in the global aging population, aortic stenosis (AS) has become one of the most common valvular diseases [1]. Surgical aortic valve replacement (SAVR) and transcatheter aortic valve implantation (TAVI) are the standard treatments for patients with severe AS. A recent meta-analysis reported that for asymptomatic patients with severe AS, the risk of all-cause mortality and heart failure (HF) re-hospitalization was significantly lower with SAVR compared to conservative treatment [2]. Data from the AVATAR trial showed that compared to conservative care, SAVR can achieve better outcomes among patients with asymptomatic severe AS, with a 54% relative lower risk for the primary composite endpoint, including acute myocardial infarction, hospitalization for heart failure, stroke, and all-cause death [3]. Despite the great advances in cardiac surgery and perioperative management, the prevention and treatment of postoperative complications is still an important component of postoperative care in cardiac surgery. However, a large proportion of patients with AS do not undergo SAVR due to various risk factors and severe surgical complications, and up to 30% of patients undergo SAVR based on the report of the Euro Heart Survey [4].

Acute kidney injury (AKI), classically characterized by decreased glomerular filtration rate, is a common complication and co-morbidity in the perioperative period of SAVR, which prolongs the length of stay in intensive care unit (ICU)/surgical intensive care unit (SICU), and hospital, increases hospitalization costs and leads to death [5–7]. The reported incidence of AKI in SAVR was higher than that after TAVI in the PARTNER (Placement of Aortic Transcatheter Valve) 2 A trial and SAPIEN 3 Intermediate Risk Registry. The results showed that among patients with severe AS and chronic kidney disease (CKD), AKI was more prevalent after SAVR than after TAVI [8]. Impaired renal function can decrease the survival rate and the quality of life of patients and there is no way to completely prevent AKI. AKI not only reduces the long-term survival rate of cardiac surgery but also increases in-hospital mortality and incurs a high cost of treatment. Therefore, it is important to determine the risk for AKI after SAVR.

Different baseline characteristics and postoperative conditions were suggested as predictors of AKI, including usually used surgical risk scores, such as the ACEF sore. This simple ACEF score combines the following three clinical variables: age, creatinine, and left ventricular ejection fraction (LVEF), which are well-recognized as independent risk factors for postoperative AKI among patients undergoing cardiac surgery. Therefore, the ACEF scoring system may be a helpful and applicable risk model for predicting postoperative AKI. The ACEF score has been assessed in patients undergoing coronary artery bypass grafting (CABG), percutaneous coronary intervention (PCI), and TAVI and has been shown to be useful in predicting outcomes [9–15]. To our knowledge, few studies have employed this risk model to predict the risk and severity of AKI after SAVR.

In this study, we aimed to investigate the risk factors of AKI after SAVR. Especially, we used the ACEF score in predicting AKI to provide a basis for reducing the risk of postoperative AKI and improving the efficacy of heart valve replacement surgery.

## Methods

### Study design

We collected the medical records of consecutive patients with symptomatic severe aortic stenosis (AS) undergoing TAVR between 1 May 2020 and 31 April 2022 in Beijing Anzhen Hospital. This study was approved by the Ethics Committee of Anzhen Hospital in Beijing, China. Severe AS was diagnosed following the ACC/AHA guideline [16]. Patients undergoing simultaneous repair of ventricular septal defect, atrial septal defect, and left ventricular aneurysm, ascending aorta replacement, and coronary artery bypass grafting were excluded. In addition, those undergoing preoperative hemodialysis or CRRT, emergency surgery, and secondary cardiac surgery before SAVR were excluded.

AKI was defined as the percentage change in estimated glomerular filtration rate (eGFR) 48 h after AVR < −25% compared to the baseline eGFR (RIFLE criteria) [17].

The ACEF score was calculated for each patient based on the preprocedural assessment and was calculated as age/left ventricular ejection fraction (LVEF) +1 (if creatinine >2.0 mg/dL) [10,18].

### Data collection

Baseline clinical characteristics of all patients were collected, including age, sex, weight, New York Heart Association Class grading IV (NYHA), presence of comorbidities (high blood pressure, diabetes, coronary heart disease, PCI, chronic obstructive pulmonary disease (COPD), and CKD), history of smoking or drinking, use of angiotensin-converting enzyme inhibitors/angiotensin receptor blocker (ACEI/ARB) and β-blocker, baseline serum creatinine level, eGFR, ultrasonic electrocardiogram data (LVEF, left interior diameter, mean gradient, aortic valve velocity), intake and output volume within 24 h, the volume of drainage within 24 h, mechanical ventilation time, ICU stay, and hospital stay were used to assess renal function.

### Statistical analysis

Continuous variables are expressed as mean values ± standard deviation (SD)/median and interquartile range (25%–75%) based on their distributions. Categorical variables are presented as frequencies (percentages). Continuous variables were compared using student’s t-test or Mann-Whitney test and categorical variables were compared using the Chi-square test. Parameters with a *p* value <.1 in univariate tests were entered into the multivariable model. Odds ratios (ORs) and 95% confidence intervals (CIs) for the association between AKI and ACEF were determined using univariate and multivariable logistic regression analyses. Parameters with a *p* value <.1 in univariate tests were entered into the multivariable model. The area under the curve (AUC), receiver operating characteristic (ROC), and Brier Score analysis were used to determine the ability of ACEF to distinguish patients with a high risk of AKI. All analyses were conducted at a 5% two-sided significance level using SPSS software version 23 (SPSS Inc., Chicago, Illinois, USA) and statistical software packages R (http://www.r-project.org, The R Foundation).

## Results

### The overall characteristics of the included patients

**Figure 1.**
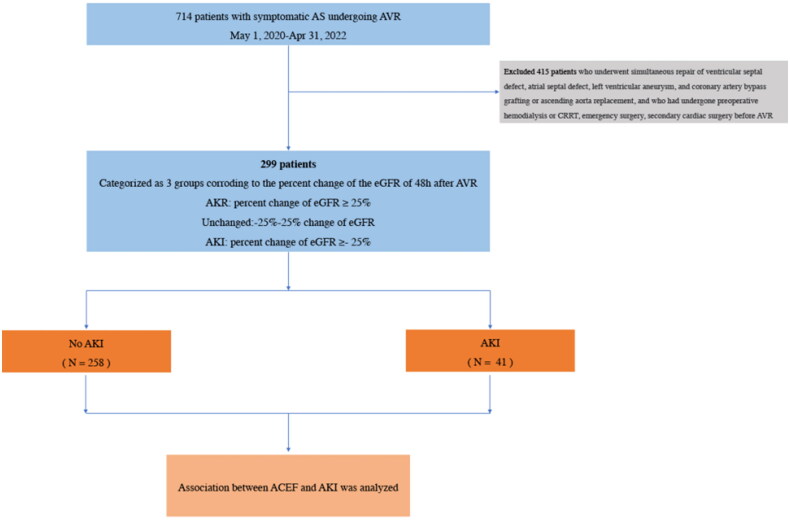
Flow chart of inclusion and exclusion process of patients.

At the beginning, 714 patients undergoing AVR were enrolled in our study. Then, patients undergoing simultaneous repair of ventricular septal defect, atrial septal defect, and left ventricular aneurysm, and coronary artery bypass grafting, or ascending aorta replacement, and those undergoing preoperative hemodialysis or CRRT, emergency surgery, and secondary cardiac surgery before AVR were excluded (Figure 1). Variations in eGFR are not accurate among these patients. Finally, we excluded 415 patients based on the exclusion criteria and the study included 299 patients. The enrolled patients were categorized into two groups based on the percentage change in eGFR. 41% of patients were assigned to the AKI group with a percentage change in eGFR ≥ − 25% within the first 48 h and 258 patients did not experience AKI.

The mean age of the study population was 59.03 years, and 52.2% (*n* = 156) of patients were men. Overall, the ACEF score was 1.07 ± 0.44. A complete list of baseline characteristics of the study population is presented in Table 1.

**Table 1.** Clinical data.

		Estimated glomerular filtration rate (eGFR) (mL/min/1.73 m²)		
	Overall (*n* = 299)	AKI (*n* = 41)	No AKI (*n* = 258)	*p* Value
Age, years	59.03 ± 10.67	56.37 ± 6.91	8.18 ± 10.67	.001
Age ≥ 65, n (%)	118 (39.5)	29 (70.7)	89 (34.50)	<.001
Age < 55, n (%)	59 (19.7)	7 (17.1)	52 (20.2)	.07
55≤ age< 65, n (%)	122 (40.8)	5 (12.2)	117 (45.3)	.41
65≤ age< 75, n (%)	83 (27.8)	26 (63.4)	57 (22.1)	<.001
75≤ age (%)	35 (11.7)	3 (7.3)	32 (12.4)	.02
Male, n (%)	156 (52.2)	19 (46.3)	137 (53.1)	.524
BMI, kg/m^2^	22.08 ± 4.78	24.50 ± 4.21	21.70 ± 4.76	<.001
Overweight, n (%)	105 (35.1)	21 (51.2)	84 (32.6)	.032
Smoking, n (%)	79 (26.4)	10 (24.4)	69 (26.7)	.899
Drinking, n (%)	41 (13.7)	5 (12.2)	36 (14.0)	.952
DMs, n (%)	153 (51.2)	23 (56.1)	130 (50.4)	.014
HTN, n (%)	119 (39.8)	24 (58.5)	95 (36.8)	.609
CHD, n (%)	29 (9.7)	5 (12.2)	24 (9.3)	.766
PCI, n (%)	9 (3.0)	3 (7.3)	6 (2.3)	.213
COPD, n (%)	110 (6.8)	17 (41.5)	93 (36)	.621
PAD, n (%)	4 (1.3)	0 (0.0)	4 (1.6)	.943
Stroke, n (%)	13 (4.3)	1 (2.4)	12 (4.7)	.816
ACEI/ARB, n (%)	54 (18.1)	13 (31.7)	41 (15.9)	.026
β-blocker, n (%)	71 (23.7)	19 (46.3)	52 (20.2)	.001
NYHA III/IV	165 (55.2)	27 (65.9)	138(53.1)	.19
Echocardiography				
LVEF (%)	61.89 (8.29)	60.90 ± 7.45	61.47 ± 8.43	.685
LVEDD, m	48.39 ± 6.85	47.98 ± 6.91	48.46 ± 6.85	.677
Mean gradient, (mmHg)	95.34 ± 29.52	100.05 ± 34.79	94.59 ± 28.60	.272
Aortic valve velocity	483.52 ± 75.06	494. 49 ± 83.40	481.77 ± 73.68	.314
ACEF	1.07 ± 0.44	1.28 ± 0.62	1.04 ± 0.39	.001
Iov 24	4,434.28 ± 1306.09	3,972.68 ± 1477.02	4,403.33 ± 1270.01	.05
Dv 24	4,728.62 ± 1176.35	4,523.46 ± 1333.96	4,761.22 ± 1148.84	.23
ICU, median [IQR]	1.00 [1.00, 2.00]	2.00 [1.00, 3.00]	1.00 [1.00, 1.00]	<.001
Ventilator time, median [IQR]	16.00 [12.00, 19.00]	17.00 [14.00, 23.00]	15.00 [12.00, 18.00]	.005
Hospital, median [IQR]	13.00 [10.00, 15.00]	14.00 [12.00, 15.00]	13.00 [10.00, 14.00]	.027
Cr	70.12 ± 14.87	69.54 ± 14.29	74.33 ± 17.69	.050
eGFR	92.73 ± 13.58	85.26 ± 11.78	93.92 ± 13.32	<.001
eGFR 24	92.36 ± 17.57	67.77 ± 17.04	96.27 ± 14.35	.016
eGFR 48	89.50 ± 20.81	54.53 ± 11.96	95.05 ± 15.93	<.001

Abbreviations: ACEI: angiotensin-converting enzyme inhibitors; ARB: angiotensin receptor blocker; PAD: percutaneous transluminal coronary intervention; COPD: chronic obstructive pulmonary disease; LVEF: left ventricular ejection fraction; NYHA: New York Heart Association; LVEDD: left ventricular end-diastolic diameter, mm; Iov 24: the volume of intake and output within 24 h after SAVR; Dv 24: the volume of drainage within 24 h after SAVR; ventime: mechanical ventilation time after SAVR; Cr 48: Cr level 48 h after SAVR; eGFR 48: eGFR 48 h after SAVR.

In total, 41 patients (13.7%) developed postoperative AKI within the first 48 h. Participants with AKI were older than their healthy counterparts. The AKI group had a higher weight and body mass index (BMI). Compared to those without AKI, participants with AKI were more likely to have diabetes, use of ACEI/ARB or β-blockers. Regarding cardiac function, the A wave was augmented in those with AKI compared to those without AKI, whereas LVEF or NYHA class did not significantly differ between the two groups. Additionally, patients with AKI had a greater intake and output volume within 24 h after AVR, drainage volume within 24 h after AVR, longer mechanical ventilation time, and longer ICU and hospital stay. Finally, the level of eGFR and ACEF scores were significantly greater in the AKI group compared to the non-AKI group.

### Predictive value of the ACEF score for AKI

**Figure 2.**
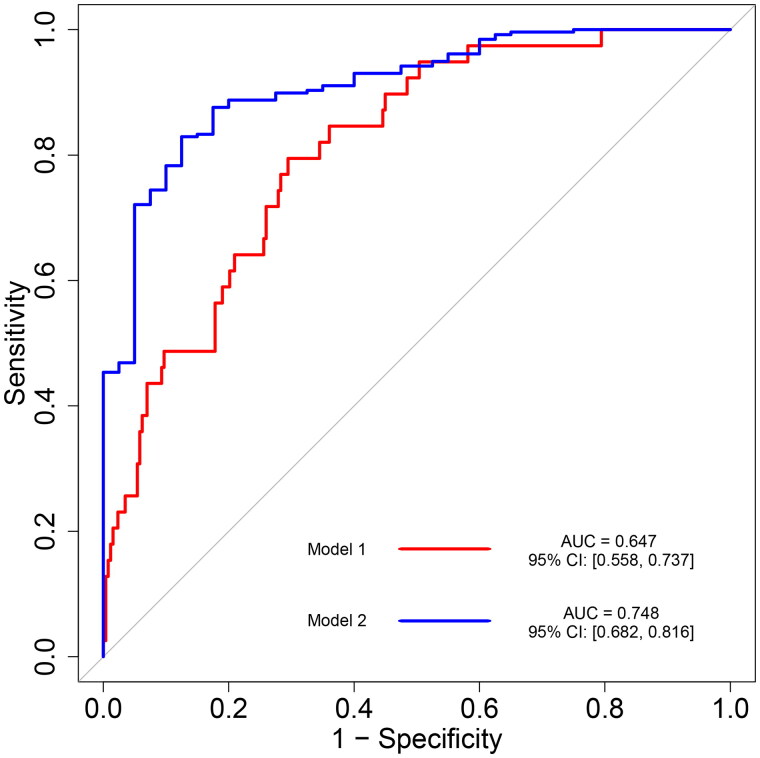
Receiver operating characteristic (ROC) curve of ACEF for acute kidney injury.

To confirm the findings of the logistic regression models were robust, we conducted a stratified analysis by subgroups based on covariates with major roles in AKI, including age, sex, BMI, overweight (BMI >24), comorbidities (HTN, DM, CHD, and COPD), history of smoking or drinking, use of ACEI/ARB and β-blocker, LVEF, ACEF scores, and mechanical ventilation time. In this model, BMI >24 (OR = 2.175 (1.116, 4.255), *p* = .022), history of hypertension (2.422 (1.246, 4.805), *p* = .009), use of ACEI/ARB (OR = 2.457 (1.147, 5.069), *p* = .017) and β-blocker (OR = 3.421 (1.715, 6.799), *p* < .001), ACEF scores (OR = 2.502 (1.348, 4.754), *p* = .004), and mechanical ventilation time 4 (3.238 (1.133, 11.809), *p* = .0045) were associated with the incidence of AKI among patients undergoing AVR (Table 2). In the univariate logistic regression, each unit of increase in the ACEF score could lead to 105.2% increase in the risk of AKI. The ROC curve was employed to assess the utility of the ACEF score for predicting AKI. The area under the ROC curve was 0.647 ± 0.46 (95% CI 0.558, 0.737). The subjects were divided into a low ACEF score group (ACEF < 1.1 and a high ACEF score group (ACEF ≥ 1.1) (Figure 2). Compared to subjects with ACEF < 1.1, subjects with ACEF ≥1.1 had a 199.1% increase in the risk of AKI. After dividing ACEF into quartiles, a 3.295-fold increase in the risk of AKI was observed when comparing the fourth quartile with the first category in the univariate logistic regression (Table 2).

**Table 2.** Univariate logistic regression model demonstrating the association between ACEF and AKI.

	OR (95% CI)	*p*
Age ≥ 75	1.588 (0.080, 11.071)	.683
Male	1.311 (0.677, 2.559)	.422
Smoking	0.8834 (0.393, 1.841)	.751
Drinking	0.856 (0.280, 2.154)	.761
BMI	2.175 (1.116, 4.255)	.022
DM	1.258 (0.650, 2.469)	.497
HTN	2.422 (1.246, 4.805)	.009
CHD	1.354 (0.435, 3.521)	.562
COPD	1.257 (0.634, 2.447)	.505
ACEI/ARB	2.457 (1.147, 5.069)	.017
β-blocker	3.421 (1.715, 6.799)	<.001
LVEF	0.992 (0.955, 1.033)	.684
ACEF	2.502 (1.348, 4.754)	.004
QACEF2	2.040 (0.667, 6.951)	.223
QACEF3	1.677 (0.551, 5.688)	.375
QACEF4	4.2945 (1.600, 13.660)	.007
ACEF cut	2.991 (1.530, 5.904)	.001
QICU2	0.548 (0.178, 1.562)	.270
QICU3	1.066 (0.421, 2.728)	.892
QICU4	1.446 (0.601, 3.590)	.414
Q Ventime2	1.463 (0.462, 5.567)	.538
Q Ventime3	1.846 (0.579, 7.052)	.324
Q Ventime4	3.238 (1.133, 11.809)	.045

Abbreviations: OR: odds ratio; 95% CI: 95% confidence interval; SD: standard deviation. Other abbreviations as in Table 1.

Multivariate logistic regression model also demonstrated the close association between the ACEF score and AKI (Table 3). After adjusting for age, BMI, history of hypertension, mechanical ventilation time, use of β-blocker and ACEI, each increment in the ACEF score led to 1.276% increase in the risk of AKI. Compared to subjects with ACEF < 1.1, subjects with ACEF ≥ 1.1 had a 179.2% increase in the risk of AKI after adjustment. However, no increase in the risk of AKI was observed in the fourth quartile of ACEF compared to the first quartile.

**Table 3.** The effect of ACEF on acute kidney injury assessed by multivariate logistic regression models.

	Odds ratio (95% CI)		Odds ratio (95% CI)	
Variables	Crude	*p* Value	Adjusted	*p* Value
ACEF (per 1 increase)	2.502 (1.348, 4.7543)	.004	2.276 (1.145, 4.598)	.019
Quartiles of ACEF				
Quartile 1	1.000 (reference)			
Quartile 2	2.04 (0.667, 6.951)	.223	1.795 (0.534, 6.620435165)	.353
Quartile 3	1.677 (0.551, 5.689)	.375	1.030 (0.301, 3.808)	.962
Quartile 4	4.295 (1.600, 13.660)	.006	3.049 (0.985, 10.806)	.064
ACEF < 1.1	1.000 (reference)			
ACEF> =1.1	2.991 (1.530, 5.904)	.001	2.792 (1.325, 5.982)	.007

Abbreviations: OR: odds ratio; 95% CI: 95% confidence interval; SD: standard deviation; Crude: no adjustment; Adjusted: adjusted for BMI, history of hypertension, mechanical ventilation time, use of β-blocker and use of ACEI; Quartile 1: ACEF< 0.860; Quartile 2: 0.860 ≤ ACEF < 0.980; Quartile 3: 0.980 ≤ ACEF < 1.145; Quartile 4: ACEF ≥ 1.145.

**Figure 3.**
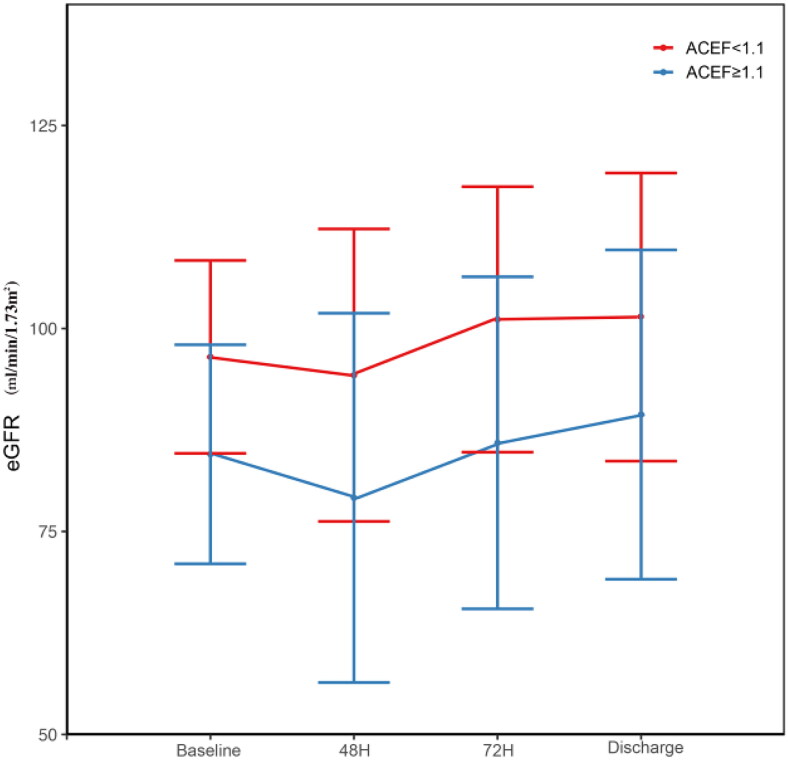
Kidney function among patients in high/low ACM group. Abbreviations: Baseline: the baseline eGFR before AVR; 48H: the eGFR of 48h after AVR; 72H: the eGFR of 72h after AVR; Discharge: the eGFR of discharged time.

**Figure 4.**
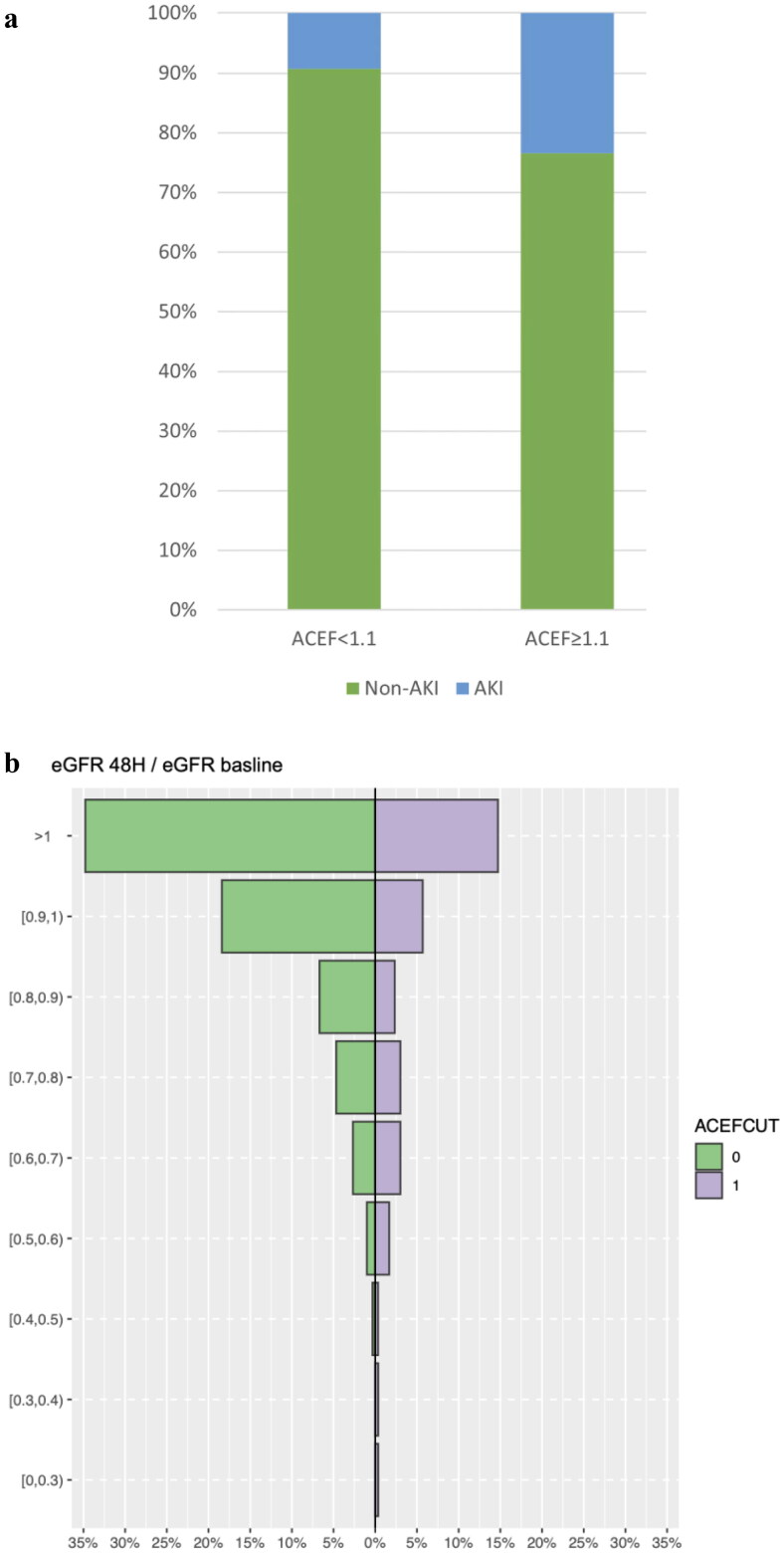
(a) Acute kidney injury among patients who underwent AVR. (b) Change of eGFR at 48 h after AVR among patients stratified by ACEF.

Furthermore, we assessed the changes in eGFR after AVR (Table 4 and Figure 3). Subjects with ACEF > = 1.1 had a lower eGFR at baseline, and 48 h after AVR,72 h after AVR, and at discharge. Our analysis showed that among those with ACEF <1.1, 19 (9.3%) had AKI and 186 (90%) did not have AKI, whereas among those with ACEF > =1.1, 22 (23.4%) had AKI and 72(76.6%) did not have AKI (Figure 4(a)). In general, patients with ACEF > =1.1 were more likely to experience AKI (Figure 4(b)).

**Table 4.** Changes in eGFR stratified by ACEF.

Level		Overall	ACEF < 1.1	ACEF> =1.1	
n		299	205	94	*p*
eGFR baseline (mean (SD)		92.73 (13.58)	96.50 (11.88)	84.51 (13.49)	<.001
eGFR 24 (mean (SD)		92.36 (70.57)	94.87 (60.24)	86.88 (89.18)	.364
eGFR 48 (mean (SD)		89.50 (20.81)	94.25 (18.01)	79.14 (22.74)	<.001
eGFR 72 (mean (SD)		96.32 (19.07)	101.12 (16.35)	85.92 (20.45)	<.001
eGFR discharge (mean (SD)		97.64 (19.36)	101.41 (17.74)	89.40 (20.28)	<.001
e 48 (mean (SD)		0.96 (0.17)	0.98 (0.15)	0.93 (0.22)	.043
AKI (%)	0	258 (86.3)	186 (90.7)	72 (76.6)	.002
	1	41 (13.7)	19 (9.3)	22 (23.4)	

## Discussion

AKI is one of the most common adverse events after SAVR, which decreases the survival rate. In recent years, the reported incidence of AKI after cardiac surgery has been reported to be 3.4% to 57% [19–21]. In the present study, we evaluated the efficacy of the ACEF score, in combination with other traditional risk factors of AKI, in predicting AKI among patients undergoing SAVR. Our major findings were as follows: (1) AKI occurred in 13.7% of patients after SAVR. (2) The ACEF score plays an important role in predicting AKI after SAVR (OR = 2.502 (1.348, 4.754), *p* = .004), along with overweight (BMI >24), the history of hypertension, the use of ACEI/ARB and β-blockers, and mechanical ventilation time. (3) ACEF score ≥ 1.11 was the optimal cutoff to identify patients who developed AKI after SAVR.

Advanced age is a risk factor for AKI after cardiac surgery. European guidelines recommend TAVI for patients aged 75 years or older, and prioritize it over SAVR. US guidelines similarly recommend TAVI over SAVR for patients aged 80 and older and suggest that they can be equally used for patients aged 65 and older [22,23]. Elderly patients are more susceptible to surgical stress, which increases the risk of AKI after cardiac surgery. The incidence of postoperative acute renal failure is higher among older adults due to renal hemodynamics and renal vasodilation in the elderly. As expected, we have identified age as a predictor of AKI after SAVR showed that older people are more prone to AKI.

Elevated preoperative Cr level is an important risk factor for postoperative AKI, and the risk of AKI increases by 4.8 times for each 1 mg/dL increase in Cr level. When the baseline creatinine concentration is 2.0–4.0 mg/dL, the risk of postoperative dialysis is 10% to 20%, and when the baseline creatinine concentration is more than 4.0 mg/dL, the risk of postoperative dialysis is nearly 25% [24]. In addition to increasing the risk of AKI, baseline creatinine levels greater than 2.5 mg/dL usually increase the risk of death and prolong the length of hospital stay after certain surgeries. However, preoperative reversible renal insufficiency does not increase the risk of postoperative AKI after cardiac valve surgery [25]. Apart from being fluid-dependent, Cr has several deficiencies as an indicator of AKI. The low muscle mass among elderly patients and frail TAVR patients reduces Cr production, which undermines the ability of Cr to reflect a true decrease in eGFR, thus leading to delayed or missed diagnosis of AKI [26]. In our study, the mean level of Cr was 70.12 ± 14.87 while the mean eGFR was 92.73 ± 13.58, suggesting that AKI also frequently occurred in patients with normal kidney function or in those with CKD1. This mean age of patients undergoing surgery was 59.03 ± 10.67 years in this study, and both Cr and eGFR were invaluable predictors of AKI. Meanwhile, the baseline, 24 h post-SVAR and 48 h post-SAVR eGFR were supposed to be all related to AKI in our study. These results confirmed that eGFR is an excellent predictor of AKI and suggested that continuous monitoring of eGFR during the perioperative period of TAVR and timely intervention may effectively prevent or delay the progression of AKI.

Aortic stenosis is a long process associated with the gradual deterioration of cardiac function over time, which is an important predictor of AKI after SAVR. In our study, we found that age and Cr were associated with AKI in patients undergoing SAVR, whereas LEVF was not associated with the development of AKI which may be due to normal LVEF in most of our patients. The ACEF score includes few variables and is easy to calculate, while also maintaining a high discriminative ability. It is a simple tool for predicting clinical outcomes in elective patients undergoing surgical revascularization [10,27], AVR or transcatheter TAVR [28]. Considering the close association between renal function and cardiac function and AKI, we hypothesized that the ACEF score can show good accuracy for predicting AKI in patients undergoing SAVR. Nonetheless, we also analyzed the connection between ACEF and AKI and found that it was independently associated with AKI in patients undergoing AVR. In the ACEF < 1.1 group, eGFR was higher at baseline, 24 h, 48 h after surgery, and before discharge, together with a higher baseline Cr. In the 1 ≤ ACEF group, eGFR was lower at all time points. Particularly, after dividing ACEF into quartiles, a 3.295-fold increase in the risk of AKI was observed when comparing the fourth quartile with the first quartile in the univariate logistic regression. Therefore, our study explored the prognostic value of ACEF and showed that the ACEF score was an independent predictor of AKI in patients undergoing SAVR. A worse eGFR predicted a higher ACEF score and a higher risk of postoperative AKI in patients undergoing SAVR.

Chang et al. found that the STS score can be used to accurately predict stage 2 and stage 3 AKI after mitral valve repair, while the ACEF score can be used to predict all stages of AKI. They showed that early management of AKI can reduce the risk of adverse events and benefit patients [29]. The ACEF score incorporates only three variables; therefore, the ACEF score is feasible for clinical use because of its simplicity and high predictive accuracy. Moreover, the ACEF risk score, which uses Cr instead of eGFR, is likely to be even simpler in the emergency department.

The conclusions of previous regarding the role of BMI in the development of kidney disease are not consistent. One study showed that obesity is associated with microalbuminuria [30]. In contrast, another study showed that morbid obesity (BMI ≥ 35 kg/m^2^) is associated with CKD, but these associations are due to the presence of diabetes and hypertension [31]. There are also studies that did not find any association between BMI and CKD [32]. The Framingham study found that baseline BMI can predict subsequent kidney disease. The study revealed that each one-unit increase in BMI can increase 1.2 the risk of new-onset kidney disease after controlling for age, sex, baseline GFR, smoking, and urine sugar disease status [33]. Consistently, we found a significantly higher risk of AKI in the BMI > 24 kg/m^2^ group than in the BMI < 24 kg/m^2^ group.

Sex is another independent patient-related risk factor for AVR-related AKI, and men are more likely to develop AKI than women after surgery [34]. In our study, there was no significant difference in sex between the AKI group and the non-AKI group, which is inconsistent with that reported by previous reports. This discrepancy may be related to sample size, regional economy, and understanding of the disease.

The relationship between HTN, DM, and AKI is well known as they are acknowledged risk factors for kidney injury [35–38]. In the present study, DM was strongly associated with the development of AKI in univariate analysis but not in multivariate analysis which is consistent with the findings of a previous study [39]. Although hypertension is known to be a risk factor for kidney failure, renal hypoperfusion and volume depletion may exacerbate renal injury [40,41], Perioperative hypotension is believed to be the main mechanism proposed for the development of AKI and vasoconstrictors impair kidney perfusion. Consistently, HTN was found to be an independent predictor of AKI after valve surgery [20,42]. HTN was not associated with the development of AKI in univariate analysis but was associated with AKI in multivariate analysis. The significant differences may be due to patients’ age, the control of HTN and the related drugs, such as ACEI/ARB and β-blockers. The rate of RAS inhibitor use (18.1%) in our study was lower than that in other studies (39.7%–58%) [43–45]. Patients taking ACEI/ARB or β blockers were shown to have increased odds of AKI, thus, consistent with our findings [46–48]. ACEI/ARB constricts efferent arterioles in glomeruli; therefore, RAS blockers cause efferent arteriolar dilatation. When the maintenance of glomerular filtration requires efferent arteriolar constriction, RAS blockers may lead to AKI [49]. Similarly, β blockers may contribute to AKI *via* hypotension with a subsequent impairment of renal perfusion during surgery due to the use of β-blockers [46]. The beneficial effects of pre-TAVR ACEI/ARB on post-TAVR survival have been previously demonstrated [50]. In patients receiving SAVR for severe AS without other significant valvular or coronary artery disease, treatment with ARB was associated with improved long-term postoperative survival, regardless of baseline characteristics [51]. However, preoperative treatment with ACEI was associated with an increased risk of mortality [52]. Debates still continue for the use of β-blockers. In patients with valvular heart disease, pre-operative treatment with β-blockers can increase the risk of postoperative adverse events [53] while the use of β-blockers can improve the survival of patients undergoing transcatheter SAVR [54]. Whether the ACEI/ARB/β-blocker regimen should be prophylactically prescribed for all patients before SAVR or only for those with medical indications and the best time to administer ACEI/ARB and β-blocker remain unclear.

Valve surgery has been shown to be a risk factor for AKI compared to CABG [37]. Rheumatic valve dysfunction usually requires a much more complex intra-cardiac operation involving valve reconstruction/replacement. In addition, valve operations always demand longer mechanical ventilation time, which is an independent risk factor for AKI. Consistent with relevant reports, we found that the duration of postoperative mechanical ventilation is strongly correlated with the prognosis of patients with AKI [55]. Longer mechanical ventilation time suggests longer ICU stays and hospital stays. Patients undergoing SAVR have 8 to 13 days length of stay [56–58]. The cost of SAVR has been substantially reduced by reducing the length of stay in operating room, intensive care unit, and hospital.

Several studies assessed the risk of AKI and sixteen studies found that TAVI is associated with a lower risk of AKI [59–61]. Nagaraja et al. found similarities between TAVI and SAVR [62]. The use of cardiopulmonary bypass (CPB) is an invasive procedure in the SAVR group. Other factors that affect the risk of AKI are associated with CPB [38,63,64], which leads to thrombin production, inflammatory response, and postoperative bleeding, all of which can lead to AKI. In addition, hemodynamic instability during SAVR, hypothermia, insufficient blood flow, and euvolemic hemodilution during open heart surgery have been considered risk factors for AKI after SAVR. From the pathophysiologic point of view, maintaining mean arterial pressure is the prerequisite for appropriate kidney perfusion and function. Besides preexisting diseases such as HTN, DM, and CKD, can lead to hemodynamic instability during SAVR [4,24–31]. In our study, the intake and output volume and the drainage volume within 24 h after SAVR were associated with the incidence of AKI. They may be associated with fluid balance, which is an important factor affecting cardiac output and renal perfusion.

## Limitations

A clear limitation of our study was its single-center design. The results of this study may not be representative of all patients undergoing AVR. Contemporary perioperative management, procedural techniques, antithrombotic regimen, and discharge management of patients undergoing SAVR varies between courtiers. Furthermore, re-hospitalization differs a lot between continents and countries. Hence, multi-center studies with more patients are needed for further verification. Furthermore, despite the use of a range of adjustment variables in the multivariate model, the effect of other confounding factors not included in this study could not be excluded. There were changes in LVEF and serum creatinine values during hospitalization. In this study, the ACEF score was only measured for the first time after admission, therefore, its dynamic changes were not monitored. While this retrospective study focused on early postoperative outcomes, the follow-up duration of seven days may be too short, and mild-to-moderate AKI may not lead to substantial chronic kidney disease. Due to the difficulties in follow-up, ACEF was not collected during the follow-up period; thus, the predictive value of the ACEF score compared to EuroSCORE or STS scores for renal function, re-hospitalization rate, and mortality remains unclear. Since the ACEF score was not statistically significant after correction, we did not find the *p* value for the trend but found that the best cutoff was 1.1. An adequately powered multi-center trial is needed to confirm our results.

## Conclusions

In conclusion, the ACEF score is a simple and effective tool for predicting AKI in patients undergoing SAVR. Our study validated that this prediction model can be easily used in clinical practice. Patients with a high risk of infection identified by the ACEF score should be considered for timely clinical intervention to reduce the incidence of complications. Further studies are needed to explore the value of the ACEF score in clinical practice and to compare its performance with other well-acknowledged and validated predictive models.

## Supplementary Material

Supplemental Material

Supplemental Material
