# Supplementary material for: Age, creatinine, and ejection fraction score is a risk factor for acute kidney injury after surgical aortic valve replacement
Source: Ren Fail. 2025 Jan 13;47(1):2444401. doi: 10.1080/0886022X.2024.2444401 (PMC11734393; doi:10.1080/0886022X.2024.2444401)
Supplement: Supplemental Material [file IRNF_A_2444401_SM4462.docx]

**Table S1. The baseline classification of kidney function.**

|  | Overall  (n=299) | AKI  (n = 41) | No AKI  (n = 258) |
| --- | --- | --- | --- |
| CKD1(n)  CKD2(n)  CKD3(n) | 191  105  3 | 22  18  1 | 169  87  2 |
