# Supplementary figures and images for: Age, creatinine, and ejection fraction score is a risk factor for acute kidney injury after surgical aortic valve replacement
Source: Ren Fail. 2025 Jan 13;47(1):2444401. doi: 10.1080/0886022X.2024.2444401 (PMC11734393; doi:10.1080/0886022X.2024.2444401)

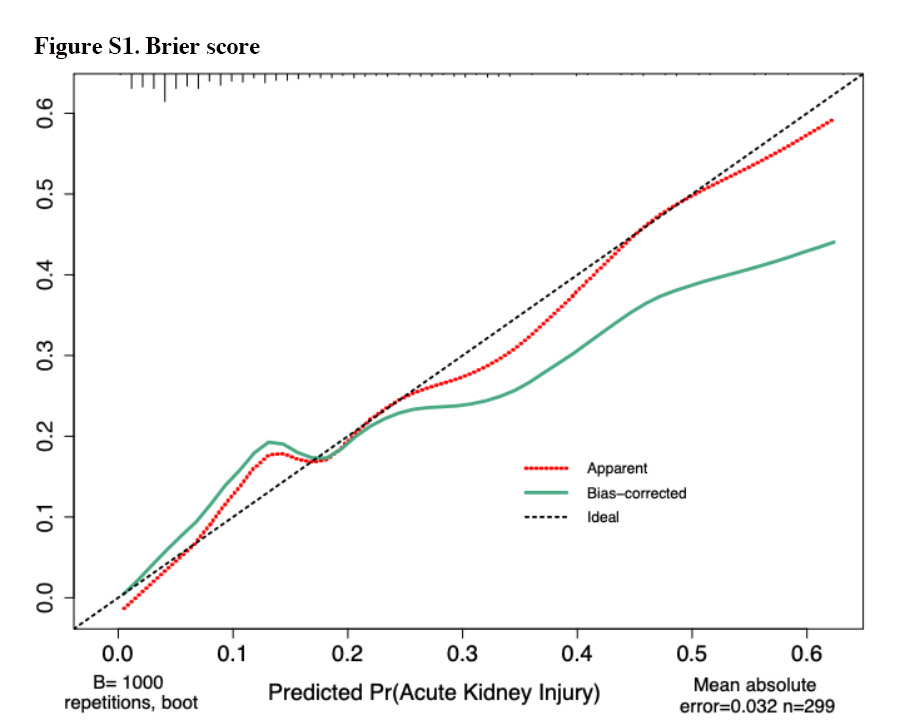

Supplement: Supplemental Material [file IRNF_A_2444401_SM4448.docx]
